# Impact of Neoadjuvant Therapy in Resected Pancreatic Ductal Adenocarcinoma of the Pancreatic Body or Tail on Surgical and Oncological Outcome: A Propensity-Score Matched Multicenter Study

**DOI:** 10.1245/s10434-019-08137-6

**Published:** 2019-12-17

**Authors:** Sanne Lof, Maarten Korrel, Jony van Hilst, Adnan Alseidi, Gianpaolo Balzano, Ugo Boggi, Giovanni Butturini, Riccardo Casadei, Safi Dokmak, Bjørn Edwin, Massimo Falconi, Tobias Keck, Giuseppe Malleo, Matteo de Pastena, Ales Tomazic, Hanneke Wilmink, Alessandro Zerbi, Marc G. Besselink, Mohammed Abu Hilal, for the European Consortium on Minimally Invasive Pancreatic Surgery (E-MIPS), Morgan Bonds, Giovanni Capretti, Guido Fiorentini, Alessandro Giardino, Carlo Lombardo, Claudio Ricci

**Affiliations:** 1 Department of Surgery, Southampton University Hospital NHS Foundation Trust, Southampton, UK; 2 Department of Surgery, Cancer Center Amsterdam, Amsterdam UMC, University of Amsterdam, Amsterdam, The Netherlands; 3 Department of Surgery, OLVG, Amsterdam, The Netherlands; 4 Department of Surgery, Virginia Mason Medical Center, Seattle, WA USA; 5 Pancreatic Surgery, San Raffaele Hospital IRCCS, Università Vita-Salute, Milan, Italy; 6 Department of Surgery, Universitá di Pisa, Pisa, Italy; 7 Department of Surgery, Pederzoli Hospital, Peschiera, Italy; 8 Department of Surgery, S. Orsola-Malpighi Hospital, Bologna, Italy; 9 Department of Surgery, Hospital of Beaujon, Clichy, France; 10 Department of Surgery, Oslo University Hospital and Institute for Clinical Medicine, Oslo, Norway; 11 Clinic for Surgery, UKSH Campus Lübeck, Lübeck, Germany; 12 Department of Surgery, Pancreas Institute, Verona University Hospital, Verona, Italy; 13 Department of Surgery, University Medical Center Ljubljana, Ljubljana, Slovenia; 14 Department of Medical Oncology, Cancer Center Amsterdam, Amsterdam UMC, University of Amsterdam, Amsterdam, The Netherlands; 15 Department of Surgery, Humanitas University Hospital, Milan, Italy; 16 Department of General Surgery, Istituto Ospedaliero Fondazione Poliambulanza, Brescia, Italy

## Abstract

**Background:**

Several studies have suggested a survival benefit of neoadjuvant therapy (NAT) for pancreatic ductal adenocarcinoma (PDAC) in the pancreatic head. Data concerning NAT for PDAC located in pancreatic body or tail are lacking.

**Methods:**

Post hoc analysis of an international multicenter retrospective cohort of distal pancreatectomy for PDAC in 34 centers from 11 countries (2007–2015). Patients who underwent resection after NAT were matched (1:1 ratio), using propensity scores based on baseline characteristics, to patients who underwent upfront resection. Median overall survival was compared using the stratified log-rank test.

**Results:**

Among 1236 patients, 136 (11.0%) received NAT, most frequently FOLFIRINOX (25.7%). In total, 94 patients receiving NAT were matched to 94 patients undergoing upfront resection. NAT was associated with less postoperative major morbidity (Clavien–Dindo ≥ 3a, 10.6% vs. 23.4%, *P* = 0.020) and pancreatic fistula grade B/C (9.6% vs. 21.3%, *P* = 0.026). NAT did not improve overall survival [27 (95% CI 14–39) versus 31 months (95% CI 19–42), *P* = 0.277], as compared with upfront resection. In a sensitivity analysis of 251 patients with radiographic tumor involvement of splenic vessels, NAT (*n* = 37, 14.7%) was associated with prolonged overall survival [36 (95% CI 18–53) versus 20 months (95% CI 15–24), *P* = 0.049], as compared with upfront resection.

**Conclusion:**

In this international multicenter cohort study, NAT for resected PDAC in pancreatic body or tail was associated with less morbidity and pancreatic fistula but similar overall survival in comparison with upfront resection. Prospective studies should confirm a survival benefit of NAT in patients with PDAC and splenic vessel involvement.

**Electronic supplementary material:**

The online version of this article (10.1245/s10434-019-08137-6) contains supplementary material, which is available to authorized users.

## Background

About 15% of cases of resectable pancreatic ductal adenocarcinoma (PDAC) are located in the pancreatic body or tail.^1^,^2^ The current standard approach in these patients is distal pancreatectomy with splenectomy followed by adjuvant chemotherapy. The vast majority (80%) of resected patients, however, will experience disease recurrence within 5 years with median overall survival of 19–32 months.^3^^–^6 A drawback of the current care is that about one-third of patients will not receive adjuvant chemotherapy following surgical resection, mostly because of poor performance status and/or surgical complications.^7^,^8^

Neoadjuvant chemo (radio) therapy (NAT) has therefore been explored as an alternative regimen which may downstage tumors leading to increased rates of R0 resection and improved survival.^7^,^9^,^10^ Furthermore a higher proportion of patients will complete NAT than adjuvant chemotherapy.^9^ Following promising initial reports concerning NAT for unresectable or locally advanced tumors only,^11^ the potential of NAT for resectable PDAC is increasingly being studied.^10^,^12^,^13^

Very few studies have addressed PDAC of the pancreatic tail or body, and the available series are mostly small, single-center reports.^14^,^15^ PDAC of the pancreatic body or tail has been shown to have distinctly different characteristics in clinical stage, vascular involvement (i.e., splenic vessel involvement), tumor biology, and gene expression when compared with tumors of the pancreatic head.^1^,^2^,^16^,^17^ Better understanding of the oncological outcomes following NAT for patients affected by PDAC of the pancreatic body or tail is therefore required.

The current study aimed to compare the clinical and oncological outcomes of NAT in patients with resected PDAC of the pancreatic body or tail with those observed in patients undergoing upfront surgery, in a multicenter propensity-score-matched cohort.

## Methods

A post hoc analysis of a previously published international multicenter retrospective cohort study of patients who underwent distal pancreatectomy for resected PDAC was performed.^18^ For the present study, the clinical and oncological outcomes of patients who underwent NAT followed by distal pancreatectomy were compared with those who received upfront surgery between 1 January 2007 and 1 July 2015. Patients with unknown NAT status, prior pancreatoduodenectomy, or metastases on initial presentation were excluded. Data on patients who may have potentially progressed or became unfit for surgery during NAT and did not proceed to surgery were not available for this study. Because of the observational study design, the need for informed consent was waived by the ethics committee of the Amsterdam UMC, location Academic Medical Center, Amsterdam.^18^ This study was conducted according to the Strengthening the Reporting of Observational Studies in Epidemiology guidelines.^19^

### Definitions

Variables included in this analysis followed the same definitions as the original DIPLOMA cohort study.^18^ NAT was defined as administration of chemotherapy, radiation, or chemoradiation before curative-intent resection. Resection margins were categorized as R0 (distance margin to tumor ≥ 1 mm), R1 (distance margin to tumor < 1 mm), or R2 (macroscopically positive margin) according to the Royal College of Pathologists definition.^20^ Clinical tumor stage was classified according to the American Joint Committee on Cancer/Union for International Cancer Control (AJCC/UICC) classification 7th edition.^21^ Postoperative morbidity was scored and classified according to the Clavien–Dindo classification.^22^ Major vascular involvement was defined as tumor involvement of any vascular structure beyond the splenic vessels on preoperative imaging. Clavien–Dindo grade III or higher complications were considered as major morbidity. Postoperative pancreatic fistula (POPF), postpancreatectomy hemorrhage (PPH), and delayed gastric emptying (DGE) were categorized according to the International Study Groups Pancreatic Surgery classification, and only grade B/C complications were considered.^23^^–^25 The 90-day mortality was noted.

### Statistical Analysis

Statistical analysis was performed using IBM SPSS^®^ Statistics for Windows version 24.0 (IBM Corp., Armonk, NY). Normally distributed variables were compared using the two-sample independent *t*-test and are reported as means with standard deviation. Non-normally distributed variables were compared using the Mann–Whitney *U* test and are presented as medians with interquartile range (IQR). Categorical variables are reported as counts with proportion and analyzed using the Chi squared or Fisher’s exact test, where appropriate.

Propensity score matching was performed, comparing patients who underwent NAT followed by resection with those who underwent upfront resection. Propensity scores were calculated by multivariable logistic regression including baseline variables age, body mass index (BMI), American Society of Anesthesiologists (ASA) physical status, tumor size, *T*-stage, and vascular involvement on preoperative imaging. With caliper width of 0.001 SD, nearest neighbors (without replacement) were matched at 1:1 ratio. To assess the balance at baseline between both groups, the standardized mean difference (SMD) was calculated, with SMD of 0.1 or below being considered to indicate optimal balance.

To analyze potential clinical selection criteria associated with administration of NAT, both univariable and multivariable binary logistic regression analyses with backward selection were performed; the results are reported as odds ratio (OR) with 95% confidence interval (CI). Variables with *p* value < 0.200 on univariable analysis or clinical relevance were selected for subsequent multivariable analysis.

Because tumor involvement of splenic vessels is regarded as a negative prognostic factor for overall survival,^26^^–^28 a sensitivity analysis was performed to investigate the impact of NAT on overall survival in patients with preoperative radiographic involvement of splenic vessels (artery and vein). A second sensitivity analysis was performed to assess the impact of different NAT regimes on overall survival.

Estimated median overall survival was calculated from date of operation to date of last follow-up or death from any cause, using Kaplan–Meier curves. The stratified log-rank test was used to compare survival distribution between groups. The level of statistical significance was set at two-sided *P* value < 0.05.

## Results

**Fig. 1:**
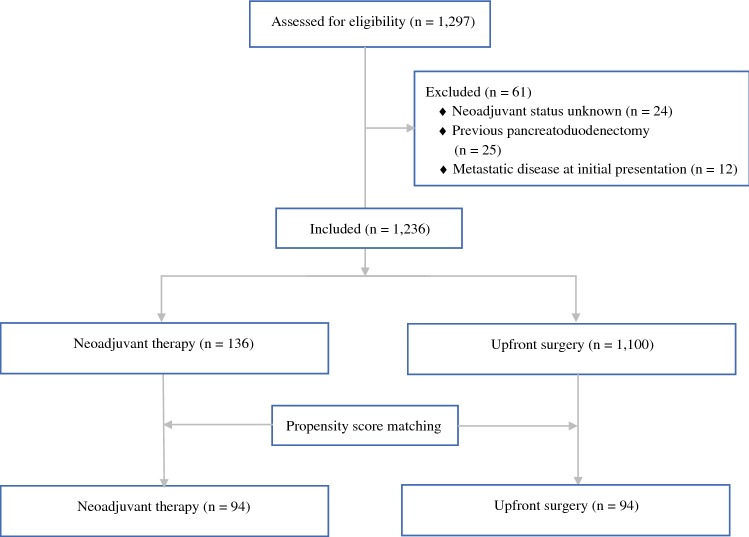
Flowchart

Among 1297 patients screened from 34 participating European and American centers, 61 patients were excluded for the reasons shown in Fig. 1. In total, 1236 patients who underwent distal pancreatectomy for PDAC were included for subsequent analysis. Overall, 136 patients (11.0%) underwent any form of NAT. Prior to matching, patients who received NAT were significantly younger (63 ± 9.6 vs. 68 ± 10.2 years, SMD − 0.39), less often classified as ASA III–IV (23.0% vs. 32.0%, SMD − 0.25), and more often underwent open distal pancreatectomy (90.4% vs. 68.7%, SMD 0.80) than those who received upfront surgery (Table 1). Tumors of patients who received NAT were smaller (30 ± 19.8 vs. 38 ± 20.8 mm, SMD − 0.39), more often located in the body of the pancreas (68.8% vs. 54.7%, SMD − 0.26), and more often involved a major vascular structure (35.7% vs. 7.7%, SMD 1.05).

**Table 1:** Baseline characteristics in both total and propensity-score-matched cohort

	Total cohort				Propensity-score-matched cohort			
	Neoadjuvant therapy (*n* = 136)	Upfront surgery (*n* = 1100)	*P* value	SMD pre	Neoadjuvant therapy (*n* = 94)	Upfront surgery (*n* = 94)	*P* value	SMD post
Age, years, mean ± SD	63 ± 9.6	68 ± 10.2	< 0.001	− 0.39	63 ± 9.5	65 ± 10.8	0.227	0.16
Age > 65, years, *n* (%)	63 (46.3)	688 (62.6)	< 0.001	− 0.36	42 (44.7)	42 (44.7)	1.000	0.00
Female sex, *n* (%)	68 (50.0)	547 (49.7)	0.952	0.01	47 (50.0)	51 (54.3)	0.559	− 0.09
Body mass index, kg/m^2^, mean ± SD	25.2 ± 4.1	25.6 ± 4.5	0.425	− 0.09	25.8 ± 3.9	26.3 ± 4.2	0.373	0.12
Unknown	16	154			11	39		
ASA classification III–IV, *n* (%)	31 (23.0)	328 (32.0)	0.032	− 0.25	22 (23.4)	30 (31.9)	0.192	− 0.23
Unknown	0	76			0	0		
Comorbidities, *n* (%)								
Prior abdominal surgery	48 (44.0)	350 (38.0)	0.221	0.13	34 (42.0)	32 (38.6)	0.655	0.07
Unknown	27	179			13	11		
Chronic pancreatitis	5 (4.0)	72 (7.2)	0.186	− 0.34	2 (2.1)	7 (7.6)	0.079	− 0.73
Unknown	11	95			0	2		
Tumor size, mm, mean ± SD	30 ± 19.8	38 ± 20.8	< 0.001	− 0.39	34 ± 19.0	29 ± 19.5	0.061	0.25
Size > 50 mm, *n* (%)	16 (12.2)	196 (18.3)	0.086	− 0.26	11 (11.7)	12 (12.8)	0.824	0.05
Unknown	4	28			0	0		
Additional organ involvement, *n* (%)	23 (18.3)	127 (12.8)	0.094	0.22	17 (18.1)	12 (12.8)	0.313	0.22
Unknown	10	111			0	0		
Major vascular involvement, *n* (%)^a^	45 (35.7)	76 (7.7)	< 0.001	1.05	19 (20.2)	20 (21.3)	0.857	− 0.03
SMV/PV	18 (14.5)	42 (4.3)	< 0.001	0.73	12 (12.8)	12 (12.8)	1.000	0.00
Truncus coeliacus	26 (21.0)	11 (1.1)	< 0.001	1.73	7 (7.4)	7 (7.4)	1.000	0.00
Other	2 (1.6)	9 (0.9)	0.465	0.31	1 (1.1)	0 (0.0)	0.500	–
Unknown	10	112			0	0		
Splenic vessel(s) involvement	23 (18.3)	189 (19.1)	0.921	− 0.01	20 (21.3)	27 (28.7)	0.238	− 0.22
Unknown	10	112			0	0		
Tumor location, *n* (%)			0.010	− 0.26			0.276	− 0.23
Body	88 (68.8)	547 (54.7)			62 (66.0)	49 (55.1)		
Tail	33 (25.8)	383 (38.3)			25 (26.6)	29 (32.6)		
Body/tail junction	7 (5.5)	70 (7.0)			7 (7.4)	11 (12.4)		
Unknown	8	100			0	5		
Procedure type, *n* (%)			< 0.001	0.80			1.000	0.00
Open DP	123 (90.4)	756 (68.7)			88 (93.6)	88 (93.6)		
Minimally invasive DP	13 (9.6)	344 (31.3)			6 (6.4)	6 (6.4)		

The specific vascular structures do not add up due to patients with involvement of more than one vascular structure

SMD on or below 0.1 was considered optimal variable balance

*ASA* American Society of Anesthesiologists, *PV* portal vein, *SMV* superior mesenteric vein, *DP* distal pancreatectomy, *SMD* standardized mean difference. SMD calculated pre and post propensity score matching

^a^Major vascular involvement beyond splenic vessels

Among those receiving NAT, chemotherapy only was provided to 106 patients (78.7%), among whom FOLFIRINOX was most frequently administered (*n* = 35, 25.7%) (Table 2). Twenty-nine patients (21.3%) received radiotherapy. Usage of NAT increased from 8.3% (*n* = 26) in 2007–2010 to 12.3% (*n* = 72) in 2013–2015 (*P* = 0.079). The proportion of FOLFIRINOX increased from 0.0% in 2007–2010 to 40.3% (*n* = 29) in 2013–2015 (*P* < 0.001).

**Table 2:** Neoadjuvant treatment

	*n* = 136
Chemotherapy, *n* (%)	133 (97.8)
FOLFIRINOX	35 (25.7)
Gemcitabine	13 (9.6)
Gemcitabine + oxaliplatin	22 (16.2)
Combination^a^	18 (13.2)
PEXG	10 (7.4)
5-fluorouracil + oxaliplatin	3 (2.2)
Unknown	32 (23.5)
Radiotherapy, *n* (%)	29 (21.3)
Only chemotherapy, *n* (%)	107 (78.7)
Combined chemotherapy and radiotherapy, *n* (%)	26 (19.1)
Only radiotherapy, *n* (%)	3 (2.2)
Total, *n*	136

PEXG includes cisplatin, epirubicin, capecitabine, and gemcitabine

^a^Including oxaliplatin with capecitabine or gemcitabine with nab-paclitaxel, cisplatin or capecitabine

### Propensity Score Matching of NAT Versus Upfront Resection

**Fig. 2:**
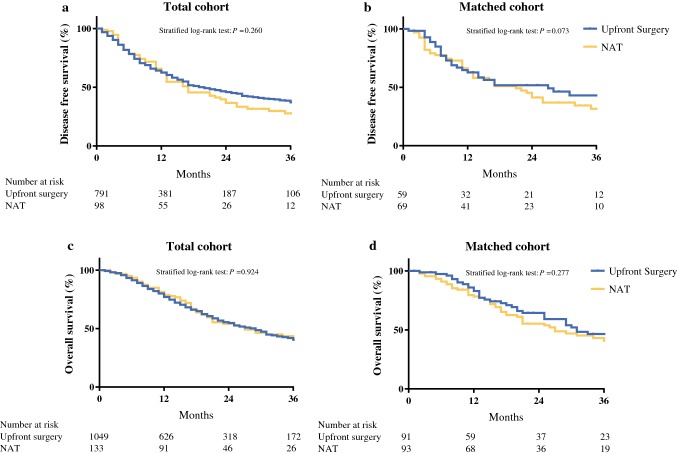
Comparison of **a**, **b** disease-free and **c**, **d** overall survival for **a**, **c** total and **b**, **d** matched cohort; NAT, neoadjuvant therapy

A total of 94 of 136 patients (69.1%) after NAT followed by surgery were matched to 94 patients who underwent upfront surgery. Forty-two patients could not be matched due to extreme baselines and missing data. After matching, baseline characteristics were well balanced (Tables 1, 3). Only the rate of patients classified as ASA III–IV remained statistical different between the NAT and upfront surgery group (23.4% vs. 30.0%, SMD − 0.23). Following surgery, patients who underwent NAT experienced less major morbidity (10.6% vs. 23.4%, *P* = 0.020), had POPF grade B/C (9.6% vs. 21.3%, *P* = 0.026), and required fewer reinterventions (6.4% vs. 19.1%, *P* = 0.009) than those who were upfront resected (Table 4). In addition, NAT was associated with fewer readmissions (5.5% vs. 18.3%, *P* = 0.008). Both groups received a comparable rate of adjuvant therapy (75.0% vs. 76.3%, *P* = 0.854). Disease-free survival and overall survival were comparable [18 (95% CI 13–22) vs. 22 months (95% CI 11–32), *P* = 0.073 and 27 (95% CI 14–39) vs. 31 months (95% CI 19–42), *P* = 0.277, respectively] between the two groups (Fig. 2).

**Table 3:** Pathology outcomes in both total and propensity-score-matched cohort

	Total cohort			Propensity-score-matched cohor		
	Neoadjuvant therapy (*n* = 136)	Upfront surgery (*n* = 1100)	*P* value	Neoadjuvant therapy (*n* = 94)	Upfront surgery (*n* = 94)	*P* value
AJCC tumor stage T3/T4, *n* (%)	86 (65.6)	846 (79.4)	< 0.001	64 (68.1)	63 (67.0)	0.876
Unknown	5	34		0	0	
Lymph node stage, *n* (%)			0.025			0.037
N0	64 (47.4)	407 (37.5)		45 (47.9)	31 (33.0)	
N1	71 (52.6)	679 (62.5)		49 (52.1)	63 (67.0)	
Unknown	1	14		0	0	
Pathological stage, *n* (%)			< 0.001			0.005
Stage 0	1 (0.7)	1 (0.1)		0 (0.0)	0 (0.0)	
Stage 1A/1B	28 (20.7)	131 (12.1)		18 (19.1)	14 (14.9)	
Stage 2A	25 (18.5)	258 (23.7)		20 (21.3)	16 (17.0)	
Stage 2B	64 (47.4)	615 (56.5)		44 (46.8)	56 (59.6)	
Stage 3	8 (5.9)	40 (3.7)		5 (5.3)	3 (3.2)	
Stage 4	9 (6.7)	43 (4.9)		7 (7.4)	5 (5.3)	
Unknown	1	12		0	0	
Lymph nodes total, median (IQR)	17 (10–25)	17 (10–26)	0.942	17 (10–27)	22 (12–35)	0.019
Lymph nodes metastatic, median (IQR)	1 (0–2)	1 (0–3)	0.003	1 (0–2)	1 (0–3)	0.101
R0 resection^a^, *n* (%)	85 (63.4)	673 (62.1)	0.761	59 (64.1)	64 (68.8)	0.500
Unknown	2	16		0	0	
Lymphovascular involvement, *n* (%)	64 (50.0)	651 (63.8)	0.002	45 (51.7)	66 (72.5)	0.004
Unknown	8	79		3	7	
Perineural involvement, *n* (%)	95 (71.4)	825 (79.8)	0.026	70 (76.1)	80 (87.9)	0.037
Unknown	3	66		2	3	

*AJCC* American Joint Committee on Cancer, 7th edition

^a^Defined as microscopic distance of ≥ 1 mm between margin and tumor

**Table 4:** Operative and postoperative outcomes in both total and propensity-score-matched cohort

Perioperative	Total cohort			Propensity-score-matched cohort		
	Neoadjuvant therapy (*n* = 136)	Upfront surgery (*n* = 1100)	*P* value	Neoadjuvant therapy (*n* = 94)	Upfront surgery (*n* = 94)	*P* value
Operative time, min, median (IQR)	255 (210–313)	240 (180–295)	0.003	255 (210–306)	229 (180–276)	0.016
Unknown	1	42		0	0	
Blood loss, ml, median (IQR)	325 (287–612)	283 (100–500)	0.017	350 (200–900)	302 (150–700)	0.214
Unknown	34	367		25	46	
Blood transfusion, *n* (%)	15 (14.3)	89 (9.8)	0.154	11 (15.7)	6 (10.7)	0.414
Unknown	31	194		24	38	
Multivisceral resection, *n* (%)	23 (18.0)	169 (16.1)	0.582	17 (18.1)	15 (16.0)	0.698
Unknown	8	48		0	0	
Vascular resection, *n* (%)	23 (16.9)	106 (9.6)	0.009	12 (12.8)	11 (11.7)	0.824
PV/SMV	71 (6.5)	14 (10.3)	0.073	8 (8.5)	8 (8.5)	1.000
Truncus coeliacus	5 (3.7)	10 (0.9)	0.018	1 (1.1)	1 (1.1)	0.751
Other	1 (0.7)	22 (2.0)	0.260	1 (1.1)	2 (2.1)	0.561
RAMPS, *n* (%)	53 (48.6)	335 (37.3)	0.022	38 (48.1)	37 (45.7)	0.759
Unknown	27	203		15	13	
Postoperative						
Major morbidity (CD ≥ 3a), *n* (%)	17 (12.5)	236 (21.5)	0.015	10 (10.6)	22 (23.4)	0.020
POPF grade B/C, *n* (%)	15 (11.0)	222 (20.2)	0.010	9 (9.6)	20 (21.3)	0.026
DGE grade B/C, *n* (%)	7 (5.2)	69 (6.4)	0.586	5 (5.3)	8 (8.5)	0.388
PPH grade B/C, *n* (%)	2 (1.5)	42 (3.9)	0.160	0 (0.0)	4 (4.3)	0.061
Surgical-site infection, *n* (%)	6 (4.5)	35(3.3)	0.488	3 (3.2)	6 (6.4)	0.249
Reintervention, *n* (%)	12 (8.8)	188 (17.1)	0.013	6 (6.4)	18 (19.1)	0.009
IC admission, *n* (%)	25 (20.2)	265 (25.8)	0.172	16 (18.4)	18 (20.0)	0.786
Hospital stay, days, median (IQR)	9 (7–14)	9 (7–14)	0.561	8 (7–12)	9 (7–12)	0.317
Readmission, *n* (%)	8 (6.3)	152 (14.8)	0.008	5 (5.5)	17 (18.3)	0.008
90-Day mortality, *n* (%)	3 (2.4)	35 (3.7)	0.467	2 (2.3)	2 (2.5)	0.923
Adjuvant therapy, *n* (%)	85 (75.9)	677 (75.1)	0.847	60 (75.0)	61 (76.3)	0.854
Unknown	24	198		14	14	
Disease-free survival, months (95% CI)	16 (12–19)	19 (15–22)	0.260	18 (13–22)	22 (11–32)	0.073
Recurrence, *n* (%)	80 (68.4)	507 (54.3)	0.004	56 (68.3)	45 (56.3)	0.114
1-Year recurrence, *n* (%)	37 (43.0)	268 (42.6)	0.942	26 (41.3)	19 (38.8)	0.789
Unknown	19	166		12	14	
Overall survival, months (95% CI)	27 (19–34)	28 (25–30)	0.924	27 (14–39)	31 (19–42)	0.277
1-Year survival, *n* (%)	91 (79.8)	626 (76.8)	0.473	68 (79.1)	59 (84.3)	0.405
3-Year survival, *n* (%)	26 (31.0)	172 (28.7)	0.665	19 (30.6)	23 (39.7)	0.301

*PV* portal vein, SMV superior mesenteric vein, *RAMPS* radical antegrade modular pancreatosplenectomy, *CD* Clavien–Dindo, *POPF* postoperative pancreatic fistula, *DGE* delayed gastric emptying, *PPH* postpancreatectomy hemorrhage, *IC* intensive care, *CI* confidence interval

### Clinical Selection Criteria for NAT

Potential selection criteria for NAT, including younger age (≤ 65 years), female sex, higher ASA classification (class III–IV), BMI, preoperative suggestion of involved major vascular structures (beyond splenic vessels), splenic vessels (artery, vein or both) or additional organ involvement, tumor size > 50 mm, and location of tumor (body, tail, or junction), were included in univariable logistic regression analysis (Supplementary Table 1). Subsequent multivariable analysis showed that age ≤ 65 years [OR 1.813 (95% CI 1.149–2.861), *P* = 0.011], major vascular involvement [OR 7.220 (95% CI 4.370–11.927), *P* < 0.001], and additional organ involvement [OR 2.027 (95% CI 1.029–3.994), *P* = 0.041] were associated with administration of NAT.

### Sensitivity Analyses

**Fig. 3:**
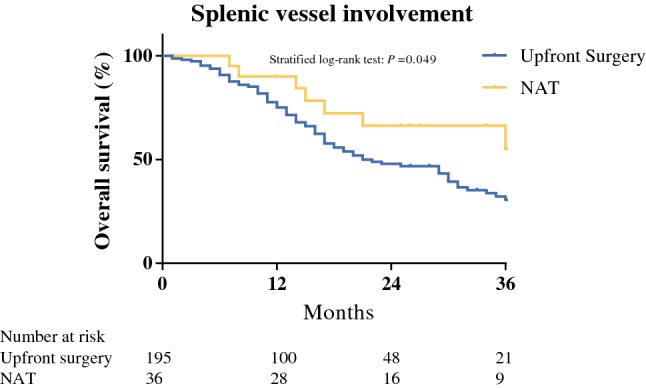
Comparison of overall survival between patients with splenic vessel involvement who underwent upfront resection or neoadjuvant therapy (NAT)

Radiologic signs of invasion of the splenic vessels on preoperative imaging were seen in 251 out of 1114 patients (22.5%), for either the splenic artery (*n* = 36, 3.2%), splenic vein (*n* = 83, 7.5%), or both (*n* = 132, 11.8%). Thirty-seven patients (14.7%) of 251 patients with splenic vessel involvement on preoperative imaging received NAT. Prolonged overall survival was found for patients with splenic vessel involvement who received NAT when compared with those who were upfront resected [36 (95% CI 18–53) vs. 20 months (95% CI 15–24), *P* = 0.049] (Fig. 3).

A second sensitivity analysis compared patients who received neoadjuvant FOLFIRINOX (*n* = 35, 25.7%) with the group who received other chemotherapy regimens. Median overall survival was statistically comparable between the two groups [41 months (95% CI median not reached) versus 26 months (95% CI 17–34), *P* = 0.095] (Supplementary Fig. 1).

## Discussion

In this international study, 136 of 1236 patients (11.0%) with resected PDAC of the pancreatic body or tail received NAT. Provision of NAT was associated with improved short-term surgical outcomes, without differences in median overall survival (27 vs. 28 months) when compared with upfront surgery. Even when balancing for potential selection factors for NAT through propensity score matching, no significant survival benefit following NAT was found (27 vs. 31 months). A potential survival benefit of NAT was observed in patients with preoperative radiologic signs of splenic vessel involvement (36 vs. 20 months). Overall survival was in line with the 19–32 months presented in previous studies concerning resected PDAC of the pancreatic body and tail.^3^^–^5

For pancreatic head cancer, both resectable and borderline resectable, several meta-analyses, large nationwide studies, and a randomized controlled trial have reported improved survival in patients who received NAT.^6^,^9^,^10^,^29^,^30^ The current study did not confirm those promising results from pancreatic head cancer. However, there is increasing evidence that PDAC in pancreatic body or tail might differ from tumors located in the pancreatic head. Besides differences in clinical stage, also tumor biology and gene expression depend on tumor location.^1^,^16^,^17^ The squamous PDAC subtype is more often found in body and tail tumors than in pancreatic head tumors and is prone to worse survival.^16^,^17^

The biological differences between tumors of the pancreatic head and body or tail might explain why the current study did not find a significant difference in overall survival following NAT. This coincides with a National Cancer Database study, including 1485 patients with resected PDAC of pancreatic body and tail, which also did not show a significant survival benefit for 176 patients who received NAT.^31^ Still, the use of NAT for body or tail tumors increased in the USA from 9.3% in 2006 to 16.9% in 2013.^31^ A comparable increase in NAT utilization from 8.3% in the period 2007–2010 to 12.3% in the period 2013–2015 was observed in the present study.

The same National Cancer Database study showed that subgroups of the PDAC population might benefit from NAT.^31^ While NAT provided for AJCC clinical stage IIa or IIb tumors was not associated with improved survival, patients with stage III disease who received NAT (*n* = 39) showed significantly prolonged overall survival compared with 49 upfront surgery patients. In the current study, the number of stage III disease patients was too limited for further analysis. Still, this highlights the importance of understanding which patients affected by PDAC might benefit most from NAT prior to surgery.

An important finding of the current study is the improved overall survival seen in patients with preoperative radiographic involvement of splenic vessels receiving NAT compared with patients with preoperative radiographic involvement of splenic vessels and undergoing upfront surgery. Although this study is the first to associate tumor involvement of the splenic vessels with administration of NAT, several previous studies have highlighted the importance of splenic vessel involvement as a negative prognostic factor for overall survival.^27^,^28^,^32^^–^34 Radiographic splenic vessel involvement is a common finding with incidence of up to 45%.^26^,^27^ While involvement of splenic artery, radiographically or pathologically, is mostly associated with reduced overall survival,^26^,^32^,^33^,^35^ an independent association between splenic vein invasion with both early liver metastasis and decreased overall survival additionally was found in another study.^34^

The significance of splenic vessel involvement for survival may be due to an increased number of circulating tumor cells in the portal vein system, as suggested by previous studies.^27^,^34^ Elevated portal vein circulating tumor cell counts are indeed associated with occurrence of liver metastases following resection 36 and decreased overall survival for patients with locally advanced PDAC.^37^ NAT appears to reduce the circulating tumor cell burden, with a preventive effect on early recurrence.^38^ The association between splenic vessel involvement and portal vein circulating tumor cells has not been established yet, and further research is needed to determine its impact on overall survival. Nonetheless, the significance of radiographic splenic vessel involvement is currently not contemplated by the centers participating in the current study, as it was not a significant selection criterion for NAT in the multivariable analysis. Hence, the current study highlights that NAT may offer oncological benefits in patients with radiographic splenic vessel involvement.

Although only 10% of surgeons considered fewer surgical complications to be a theoretical advantage of NAT in a recent survey,^39^ the current study found a significant decrease of both major morbidity and POPF grade B/C rate following NAT. The association between NAT and lower incidence of complications, especially POPF, has been highlighted by previous studies.^14^,^15^,^40^ Histopathological assessment of distal pancreatectomy specimens has shown that NAT induces lobular atrophy and fibrosis and that mainly acinar cells (i.e., exocrine function of the pancreas) were damaged by NAT.^15^,^41^ Both histopathological characteristics following NAT may influence the surgical outcome due to changes of gland texture and exocrine function of the pancreas. Indeed, in two studies, hard gland texture was more frequently observed in patients receiving NAT, and this was associated with a reduced POPF rate.^40^,^42^

The results of this study should be interpreted in light of some limitations. First, due to its retrospective design, this study compared oncological outcomes in patients who successful proceeded to resection after NAT, but did not include outcomes of patients who may have potentially progressed or became unfit during NAT. A meta-analysis of 35 studies on NAT for pancreatic cancer showed that around 18% of NAT patients did not proceed to surgery.^9^ Unfortunately, these data cannot be retrospectively retrieved and may have introduced survival bias for the NAT group. Second, there was heterogeneity in NAT regimens among centers. Owing to the low numbers per therapeutic NAT regime, the current study was not able to provide evidence on the benefits per regime. FOLFIRINOX, however, seems to be the most effective treatment of choice,^11^,^43^ and its use increased over the years in the current study. Third, the number of patients with splenic vessel involvement who underwent NAT was low. Although the difference in overall survival was significant, further research is required to assess the relevance of this finding. Fourth, due to rigid inclusion criteria and missing baseline characteristics, 42 NAT patients could not be matched to an upfront surgery patient. Still, the baseline characteristics of both groups were well balanced.

In conclusion, for patients with resected PDAC of the pancreatic body and tail, provision of NAT was associated with improved short-term surgical outcomes but did not seem to improve overall survival. However, for patients with pancreatic cancer radiologically involving the splenic vessels on preoperative imaging, NAT may improve overall survival. Future randomized controlled studies are required to determine the role of NAT in patients affected by resectable PDAC of the pancreatic body and tail, and these studies should stratify by presence of splenic vessel involvement.

## Electronic supplementary material

Below is the link to the electronic supplementary material.

Supplementary material 1 (DOCX 48 kb)

## References

[1] van Erning FN, Mackay TM, van der Geest LGM (2018). Association of the location of pancreatic ductal adenocarcinoma (head, body, tail) with tumor stage, treatment, and survival: a population-based analysis. Acta Oncol (Madr)..

[2] Artinyan A, Soriano PA, Prendergast C, Low T, Ellenhorn JDI, Kim J (2008). The anatomic location of pancreatic cancer is a prognostic factor for survival. HPB..

[3] Magge D, Gooding W, Choudry H (2013). Comparative effectiveness of minimally invasive and open distal pancreatectomy for ductal adenocarcinoma. JAMA Surg..

[4] Ruess DA, Makowiec F, Chikhladze S (2015). The prognostic influence of intrapancreatic tumor location on survival after resection of pancreatic ductal adenocarcinoma visceral and general surgery. BMC Surg..

[5] Winer LK, Dhar VK, Wima K (2019). The impact of tumor location on resection and survival for pancreatic ductal adenocarcinoma. J Surg Res..

[6] Gillen S, Schuster T, Büschenfelde CM Zum, Friess H, Kleeff J. Preoperative/neoadjuvant therapy in pancreatic cancer: a systematic review and meta-analysis of response and resection percentages. *PLoS Med*. 2010;7(4):1**–**15. 10.1371/journal.pmed.1000267. 10.1371/journal.pmed.1000267 PMC2857873 20422030

[7] Verma V, Li J, Lin C (2016). Neoadjuvant therapy for pancreatic cancer: systematic review of postoperative morbidity, mortality, and complications. Am J Clin Oncol Cancer Clin Trials..

[8] Mackay TM, Smits FJ, Roos D (2019). The risk of not receiving adjuvant chemotherapy after resection of pancreatic ductal adenocarcinoma: a nationwide analysis. HPB..

[9] Versteijne E, Vogel JA, Besselink MG (2018). Meta-analysis comparing upfront surgery with neoadjuvant treatment in patients with resectable or borderline resectable pancreatic cancer. Br J Surg..

[10] Mokdad AA, Minter RM, Zhu H (2017). Neoadjuvant therapy followed by resection versus upfront resection for resectable pancreatic cancer: a propensity score matched analysis. J Clin Oncol..

[11] Petrelli F, Coinu A, Borgonovo K (2015). FOLFIRINOX-based neoadjuvant therapy in borderline resectable or unresectable pancreatic cancer: a meta-analytical review of published studies. Pancreas..

[12] Golcher H, Brunner TB, Witzigmann H (2015). Neoadjuvant chemoradiation therapy with gemcitabine/cisplatin and surgery versus immediate surgery in resectable pancreatic cancer. Strahlenther Onkol..

[13] Tajima H, Ohta T, Kitagawa H (2012). Pilot study of neoadjuvant chemotherapy with gemcitabine and oral S-1 for resectable pancreatic cancer. Exp Ther Med..

[14] Denbo JW, Bruno ML, Cloyd JM (2016). Preoperative chemoradiation for pancreatic adenocarcinoma does not increase 90-day postoperative morbidity or mortality. J Gastrointest Surg..

[15] Takahashi H, Ogawa H, Ohigashi H (2011). Preoperative chemoradiation reduces the risk of pancreatic fistula after distal pancreatectomy for pancreatic adenocarcinoma. Surgery..

[16] Dreyer SB, Jamieson NB, Upstill-Goddard R (2018). Defining the molecular pathology of pancreatic body and tail adenocarcinoma. Br J Surg..

[17] Birnbaum DJ, Bertucci F, Finetti P, Birnbaum D, Mamessier E (2019). Head and body/tail pancreatic carcinomas are not the same tumors. Cancers (Basel)..

[18] van Hilst J, de Rooij T, Klompmaker S (2019). Minimally Invasive versus Open Distal Pancreatectomy for Ductal Adenocarcinoma (DIPLOMA). Ann Surg..

[19] Von Elm E, Altman DG, Egger M, Pocock SJ, Gøtzsche PC, Vandenbroucke JP (2007). The Strengthening the Reporting of Observational Studies in Epidemiology (STROBE) statement: guidelines for reporting observational studies. Lancet..

[20] Campbell F, Cairns A, Duthie F, Feakins R. Dataset for the histopathological reporting of carcinomas of the pancreas, ampulla of Vater and common bile duct from the Royal College of Pathologists. https://www.rcpath.org/uploads/assets/34910231-c106-4629-a2de9e9ae6f87ac1/g091-pancreasdataset-mar17.pdf. Published 2017. Accessed October 3, 2018.

[21] Edge SB, Compton CC (2010). The American Joint Committee on Cancer: The 7th edition of the AJCC cancer staging manual and the future of TNM. Ann Surg Oncol..

[22] Clavien PA, Barkun J, De Oliveira ML (2009). The Clavien-Dindo classification of surgical complications: five-year experience. Ann Surg..

[23] Wente MN, Bassi C, Dervenis C (2007). Delayed gastric emptying (DGE) after pancreatic surgery: a suggested definition by the International Study Group of Pancreatic Surgery (ISGPS). Surgery..

[24] Wente MN, Veit JA, Bassi C (2007). Postpancreatectomy hemorrhage (PPH)-An International Study Group of Pancreatic Surgery (ISGPS) definition. Surgery..

[25] Bassi C, Dervenis C, Butturini G (2005). Postoperative pancreatic fistula: an international study group (ISGPF) definition. Surgery..

[26] Takahashi H, Akita H, Gotoh K (2015). Preoperative gemcitabine-based chemoradiation therapy for pancreatic ductal adenocarcinoma of the body and tail: Impact of splenic vessels involvement on operative outcome and pattern of recurrence. Surgery..

[27] Hyun JJ, Rose JB, Alseidi AA (2019). Significance of radiographic splenic vessel involvement in the pancreatic ductal adenocarcinoma of the body and tail of the gland. J Surg Oncol..

[28] Crippa S, Cirocchi R, Maisonneuve P (2018). Systematic review and meta-analysis of prognostic role of splenic vessels infiltration in resectable pancreatic cancer. Eur J Surg Oncol..

[29] de Geus SWL, Eskander MF, Bliss LA (2017). Neoadjuvant therapy versus upfront surgery for resected pancreatic adenocarcinoma: a nationwide propensity score matched analysis. Surgery..

[30] Jang JY, Han Y, Lee H (2018). Oncological benefits of neoadjuvant chemoradiation with gemcitabine versus upfront surgery in patients with borderline resectable pancreatic cancer: a prospective, randomized, open-label, multicenter phase 2/3 trial. Ann Surg..

[31] Nelson DW, Chang SC, Grunkemeier G (2018). Resectable distal pancreas cancer: time to reconsider the role of upfront surgery. Ann Surg Oncol..

[32] Fukami Y, Kaneoka Y, Maeda A, Takayama Y, Onoe S (2016). Prognostic impact of splenic artery invasion for pancreatic cancer of the body and tail. Int J Surg..

[33] Partelli S, Crippa S, Barugola G (2011). Splenic artery invasion in pancreatic adenocarcinoma of the body and tail: a novel prognostic parameter for patient selection. Ann Surg Oncol..

[34] Mizumoto T, Toyama H, Asari S (2018). Pathological and radiological splenic vein involvement are predictors of poor prognosis and early liver metastasis after surgery in patients with pancreatic adenocarcinoma of the body and tail. Ann Surg Oncol..

[35] Kanda M, Fujii T, Sahin TT (2010). Invasion of the splenic artery is a crucial prognostic factor in carcinoma of the body and tail of the pancreas. Ann Surg..

[36] Bissolati M, Sandri MT, Burtulo G, Zorzino L, Balzano G, Braga M (2015). Portal vein-circulating tumor cells predict liver metastases in patients with resectable pancreatic cancer. Tumor Biol..

[37] Liu X, Li C, Li J (2018). Detection of CTCs in portal vein was associated with intrahepatic metastases and prognosis in patients with advanced pancreatic cancer. J Cancer..

[38] Gemenetzis G, Groot VP, Yu J (2018). Circulating tumor cells dynamics in pancreatic adenocarcinoma correlate with disease status. Ann Surg..

[39] Heinrich S, Besselink M, Moehler M (2019). Opinions and use of neoadjuvant therapy for resectable, borderline resectable, and locally advanced pancreatic cancer: international survey and case-vignette study. BMC Cancer..

[40] Zettervall SL, Ju T, Holzmacher JL, Rivas L, Lin PP, Vaziri K (2018). Neoadjuvant radiation is associated with fistula formation following pancreaticoduodenectomy. J Gastrointest Surg..

[41] Chatterjee D, M.H. K, Rashid A, et al. Pancreatic intraepithelial neoplasia and histologic changes in non-neoplastic pancreas associated with neoadjuvant therapy in patients with pancreatic ductal adenocarcinoma. *Histopathology*. 2013;63(6):841**–**851. doi:10.1111/his.12234. 10.1111/his.12234 PMC3836824 24111684

[42] Cools KS, Sanoff HK, Kim HJ, Yeh JJ, Stitzenberg KB (2018). Impact of neoadjuvant therapy on postoperative outcomes after pancreaticoduodenectomy. J Surg Oncol..

[43] Suker M, Beumer BR, Sadot E (2016). A patient-level meta-analysis of FOLFIRINOX for locally advanced pancreatic cancer. Lancet..

