# Supplementary material for: Impact of Neoadjuvant Therapy in Resected Pancreatic Ductal Adenocarcinoma of the Pancreatic Body or Tail on Surgical and Oncological Outcome: A Propensity-Score Matched Multicenter Study
Source: Ann Surg Oncol. 2019 Dec 17;27(6):1986–96. doi: 10.1245/s10434-019-08137-6 (PMC7210228; doi:10.1245/s10434-019-08137-6)
Supplement: Supplementary file 1 — Supplementary material 1 (DOCX 48 kb) [file 10434_2019_8137_MOESM1_ESM.docx]

| **Supplemental table 1.** **Univariable and multivariable analysis of clinical selection criteria associated with the administration of neoadjuvant therapy** | | | | | | |
| --- | --- | --- | --- | --- | --- | --- |
|  | | | **Univariable analysis** | | **Multivariable analysis** | |
| Factors: | | | OR (95% CI) | P-value | OR (95% CI) | P-value |
| Age | | |  |  |  |  |
|  | >65 | | Ref |  | Ref |  |
|  | ≤65 years | | 1.940 (1.355-2.776) | <0.001 | 1.813 (1.149-2.861) | 0.011 |
| Sex | | |  |  |  |  |
|  | male | | Ref |  |  |  |
|  | female | | 1.011 (0.708-1.444) | 0.952 | Removed step 4 |  |
| ASA classification | | |  |  |  |  |
|  | I-II | | Ref |  |  |  |
|  | III-IV | | 0.633 (0.415-0.965) | 0.033 | Removed step 3 |  |
| Body mass index (continuously) | | | 0.982 (0.940-1.026) | 0.420 | Removed step 2 |  |
| Preoperative imaging showing | | |  |  |  |  |
|  | Major vascular involvement^a^ | | 6.900 (4.481-10.624) | <0.001 | 7.220 (4.370-11.927) | <0.001 |
|  | Splenic vessel involvement | | 0.906 (0.557-1.474) | 0.691 | Removed step 6 |  |
|  | Additional organ involvement | | 1.820 (1.100-3.010) | 0.020 | 2.027 (1.029-3.994) | 0.041 |
|  | Tumor size | |  |  |  |  |
|  |  | ≤50mm | Ref |  | Ref |  |
|  |  | >50mm | 0.497 (0.337-0.734) | <0.001 | 0.478 (0.208-1.101) | 0.083 |
| Location tumor | | |  |  | Removed step 5 |  |
|  | Pancreatic body | | Ref |  |  |  |
|  | Pancreatic tail | | 0.536 (0.352-0.816) | 0.004 |  |  |
|  | Junction body/tail | | 0.622 (0.277-1.396) | 0.249 |  |  |
|  | | |  |  |  |  |
| *OR* indicates odds ratio, *CI* Confidence interval, *ASA* American Society of Anesthesiologists. ^a^Tumor involvement of vascular structures beyond splenic vessel involvement. | | | | | | |

**Supplemental figure 1.** Comparison of overall survival for FOLFIRINOX versus other chemotherapy regimes.
